# The limited use of autologous hematopoietic stem cell transplant for fit older patients with multiple myeloma in India: a retrospective analysis

**DOI:** 10.1186/s43046-022-00123-6

**Published:** 2022-05-16

**Authors:** Swaroop Revannasiddaiah, Prathap Raja Varma Muppalla Ayalgaraj Nagaraju, Rasmi Palassery, Apoorva Jagadish, Vinayak V. Maka, Nalini Kilara, Damiano Rondelli, Santhosh Kumar Devadas

**Affiliations:** 1 Department of Medical Oncology, Ramaiah Medical College, Bengaluru, India; 2 Department of Regulatory Affairs—Drugs, Biologics and Medical Devices, College of Professional Studies, Northeastern University, MB Boston, USA; 3 Department of Pediatrics, Louisiana State University Health Sciences Centre, Sheveport, USA; 4 Department of Hematology and Oncology, University of Illinois College of Medicine, Chicago, USA

**Keywords:** Multiple myeloma fitness assessment, Age and fitness, Autologous hematopoietic transplant in older patients, Geriatric myeloma, Autologous transplant

## Abstract

**Background:**

Multiple myeloma (MM) predominantly affects older patients; many of whom do not undergo autologous hematopoietic stem cell transplant (AHSCT) despite the associated survival benefits. This study was conceived to investigate the patterns of AHSCT among MM patients with due regard to their age and standardized fitness assessments.

**Methods:**

Fitness scores as per the hematopoietic stem cell transplant-comorbidity index (HSCT-CI) and risk scores as per the revised-myeloma comorbidity index (R-MCI) of MM patients treated between January 2017 and December 2019 were analyzed to assess fitness for AHSCT. Proportions of patients who underwent AHSCT were calculated with regard to age and fitness for AHSCT.

**Results:**

Of the 81 eligible patient records with a median age of 62 years, the HSCT-CI classified 79.6% and 77.8% of patients aged ≤65 years and >65 years as AHSCT eligible (p 1). Using the R-MCI, 96.3% and 81.5% of patients aged ≤65 years and >65 years, respectively, were classified as eligible for AHSCT (*p* 0.0381). Overall, patients aged ≤65 years underwent AHSCT with a greater frequency compared to those aged >65years (38.9 vs. 14.8%, *p* 0.0402). Irrespective of the age group, there was a statistically significant difference (*p* 0.0167) in terms of survival which favored those who underwent AHSCT.

**Conclusions:**

Both the HSCT-CI and the R-MCI revealed that nearly 80% of patients aged >65 years were fit enough to receive AHSCT. However, far fewer patients of this age group underwent AHSCT. We propose that the routine inclusion of objective fitness assessment could ensure that fit older patients undergo AHSCT and thus do not miss out on the benefits of the same.

## Background

Continuous advances in therapy have rendered MM into a “chronic disease” in recent times, though a cure is still an aspirational goal. High-dose melphalan followed by autologous hematopoietic stem cell transplant (AHSCT) remains an important part of optimal myeloma management [1]. A significant proportion of eligible MM patients do not undergo a stem cell transplant with causes including a lack of resources (especially in low-/middle-income countries) or a perceived lack of fitness (all around the world) [2–5].

Age is not to be regarded as the sole determinant of “fitness” for an AHSCT. Indices such as the “Hematopoietic Stem Cell Transplantation comorbidity index” (HSCT-CI) have been developed to be utilized as an objective guide to help in identifying fit patients for transplant [6]. There still appears to be a general bias against the older patient, and many do not undergo an AHSCT just based on age, without a formal fitness evaluation ever being done. Myeloma primarily is a disease of advanced age, and using “age” alone as a surrogate for fitness could unjustifiably disqualify a majority of MM patients from receiving an AHSCT [7–9].

The cost of transplant in a low- and middle-income country (LMIC) is often regarded as a major reason due to which a significant proportion of MM patients eligible for AHSCT fail to undergo the procedure. However, a bias against advanced age could also be contributory to the low rates of AHSCT among those eligible. This retrospective study was conceived to evaluate the impact of age upon the patterns of AHSCT among myeloma patients.

## Methods

The study was submitted to and approved by the institutional ethics committee. This retrospective study included all consecutive MM patients treated in the hospitals affiliated with our institution from January 2017 to December 2019. The hospital records of these patients were utilized to collect data regarding age and patterns of AHSCT. All patients who underwent AHSCT had received high-dose melphalan as the conditioning regimen. Stem cell mobilization was with granulocyte colony-stimulating factor (G-CSF) (and plerixafor if required). All patients who underwent transplant had signed consent forms as part of the transplant-unit policy.

Data regarding fitness was collected for each patient as per the Hematopoietic Stem Cell Transplant-Comorbidity Index (HSCT-CI), Revised-Myeloma Comorbidity Index (R-MCI), and the Charlson Comorbidity Index (CCI) [10, 11]. Data collected for HSCT-CI pertained to the presence, absence, and/or the severity of arrhythmia, cardiovascular comorbidity, inflammatory bowel disease, diabetes, cerebrovascular disease, psychiatric disturbance, hepatic comorbidity, infection, rheumatologic comorbidity, peptic ulcer disease, renal comorbidity, pulmonary comorbidity, prior history of solid tumors, heart valve disease, and the type of transplant (autologous versus allogeneic) as input parameters. The data collected for the R-MCI included information regarding creatinine clearance, pulmonary function, Karnofsky performance score, frailty, age in years, and cytogenetics as input parameters. For the CCI, data collected included age, history of myocardial infarction, congestive heart failure, peripheral vascular disease, cerebrovascular accident, dementia, chronic obstructive pulmonary disease, connective tissue disease, peptic ulcer disease, liver disease, diabetes mellitus, hemiplegia, chronic kidney disease, solid tumors, leukemia, lymphoma, and the acquired immunodeficiency syndrome.

Scoring for each of these indices was performed as per the respective validated scoring systems [10, 11]. Patients with low risk (scores of 0–3) and intermediate risk (scores of 4–6) as per the R-MCI were regarded as “fit”, “intermediate-fit,” and “unfit,” respectively. Patients with HSCT-CI scores of 0, 1–2, and >2 were regarded as fit, intermediate-fit, and unfit, respectively. As regards the CCI, scores of 0, 1, and >1 were classified as fit, intermediate fit, and unfit, respectively. In addition, descriptive data was collected regarding whether patients were offered AHSCT and whether AHSCT was done.

Survival curves were compared using the log-rank test. Proportions were compared using Fisher’s exact test. All p values are two-tailed, with the significance cut-off set at <0.05. Data entry and charting were performed using *Gnumeric* (version 1.10), and the statistical analysis was performed using *PSPP* (version 1.4). Both software are free and open-source, available under the *Gnu General Public License* (GPLv3).

## Results

**Fig. 1:**
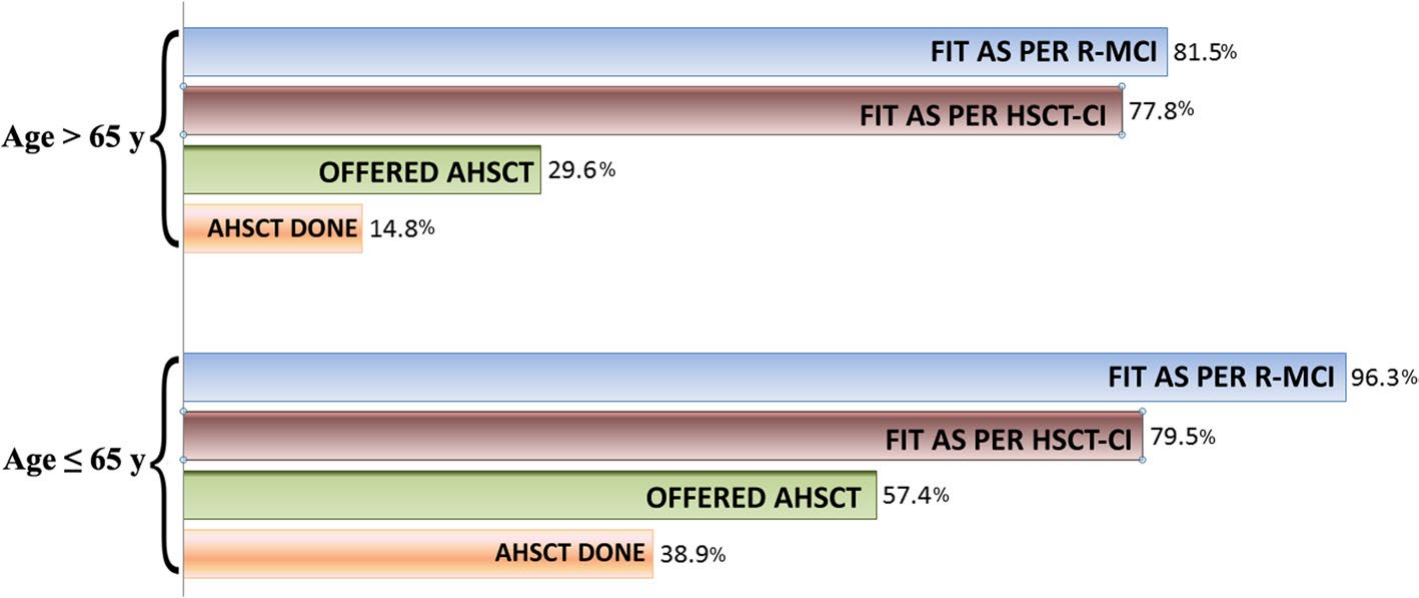
Among those aged >65 years, 81.5% and 77.8% were fit for autologous hematopoietic stem cell transplant (AHSCT) as per the Revised-Myeloma Comormibidity Index (R-MCI) & the Hematopoietic Stem Cell Transplant-Comorbidity Index (HSCT-CI), respectively. But only 29.6% were offered AHSCT, and 14.8% underwent AHSCT. In comparison, 96.3% and 79.5% of patients aged up to 65 years were fit for AHSCT as per the R-MCI & the HSCT-CI, respectively. While 57.4% were offered AHSCT and 38.9% underwent AHSCT

A total of 81 patients (54 males and 27 females) with MM were treated in our institute from January 2017 to December 2019; the patient characteristics are represented in Table 1. Evaluation of hospital records revealed that the option of AHSCT was offered to 39 patients (48.1%) and not offered to 42 patients (51.9%). Among the 39 patients who were offered AHSCT, 25 patients underwent the procedure. The median age of the overall sample of 81 patients was 62 years, with 54 (66.7%) aged ≤65 years and 27 (33.3%) aged >65 years. Those aged ≤65 years were offered AHSCT more frequently than those aged >65 years (57.4% vs. 29.6%, *p* 0.0207). Those aged ≤ 65 years underwent AHSCT more frequently than those aged > 65 years (38.9 vs. 14.8%, *p* 0.0402) (Fig. 1).

**Table 1:** Comparison of baseline characteristics, fitness patterns, and proportion of patients transplanted

	Age ≤65 years	Age >65 years	*p* value
Number of patients	54	27	-
Male to female ratio	1.84	2.37	*0.43*
Age range (in years)	38–65	66–79	-
Median age (in years)	55	72	-
Mean HSCT-CI score	1.3	1.6	*0.34*
AHSCT fit by HSCT-CI	79.6%	*77.8%*	*1*
Mean RMCI score	2.5	4.9	*<0.001*
AHSCT fit by R-MCI	96.3%	81.5%	*0.038*
Offered AHSCT	57.4%	29.6%	*0.02*
Percent Transplanted	38.9%	14.8%	*0.04*

Legend: *HSCT-CI* Hematopoietic Stem Cell Transplant Comorbidity Index, *AHSCT* autologous hematopoietic stem cell transplant, *R-MCI* Revised Myeloma Comorbidity Index

Both indices classified around four out of five (approximately 80%) MM patients as being AHSCT eligible, irrespective of the age group (Fig. 1). The R-MCI showed that more patients in the age group of ≤ 65 years were AHSCT eligible compared to those aged >65 years (96.3 vs. 81.5%, *p* 0.0381). The HSCT-CI classified a similar proportion of patients aged ≤ 65 years and > 65 years as AHSCT eligible (79.6 vs. 77.8%, p 1). The CCI classified 75.3% (61) of overall patients, and all patients aged >65 years as unfit for AHSCT. Further data analysis about the CCI was not performed.

The median duration of follow-up for the population was 21 months. The median overall survival (OS) of patients who were offered AHSCT was greater than those who were not offered transplants (median OS not reached vs. 36 months; *p* 0.033). Of those who were offered a transplant 14 did not undergo the procedure for the following reasons: concern of toxicity (five), financial (three), and unknown reasons (six patients). When analyzing the outcome of those who did or did not receive AHSCT, transplanted patients had a significantly longer OS than those who did not undergo AHSCT (median OS not reached vs. 36 months; *p* 0.0023).

**Fig. 2:**
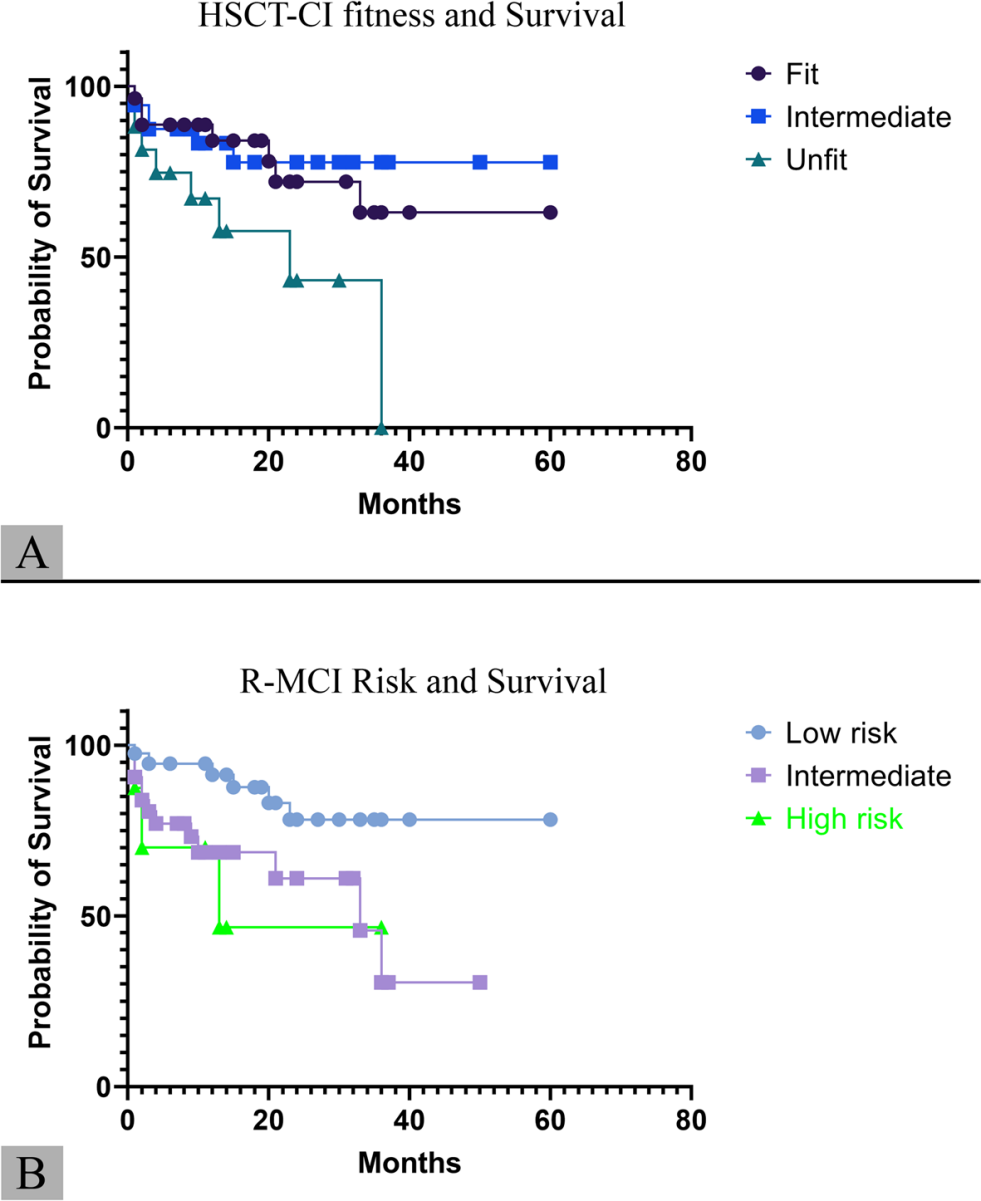
Survival curves revealing significant differences as per the Hematopoietic Stem Cell Transplant Comorbidity Index (HSCT-CI) fitness scores (*p* 0.0362) (**A**) & the Revised-Myeloma Comorbidity Index (R-MCI) risk scores (*p* 0.0253) (**B**)

While the median OS of patients classified as fit and intermediate fit as per the HSCT-CI was not reached, the OS of those classified as unfit was significantly lower at 23 months (*p* 0.0362). In addition, the median OS of patients classified as low risk by the R-MCI was not reached, while the MS of those classified as intermediate risk and high risk was significantly lower at 36 months (*p* 0.0253) (Fig. 2).

**Fig. 3:**
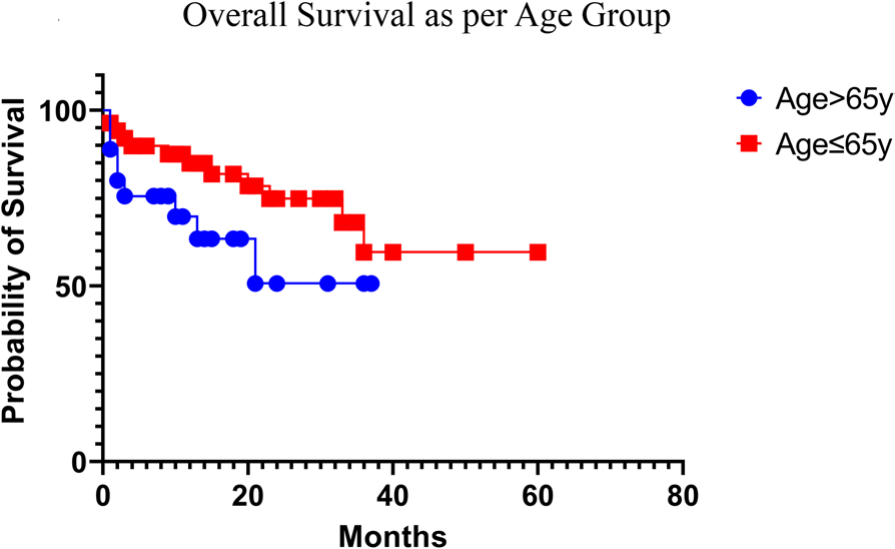
Survival curves for those aged up to and beyond 65 years of age. The median survival is not reached in both groups (*p* 0.0875)

**Fig. 4:**
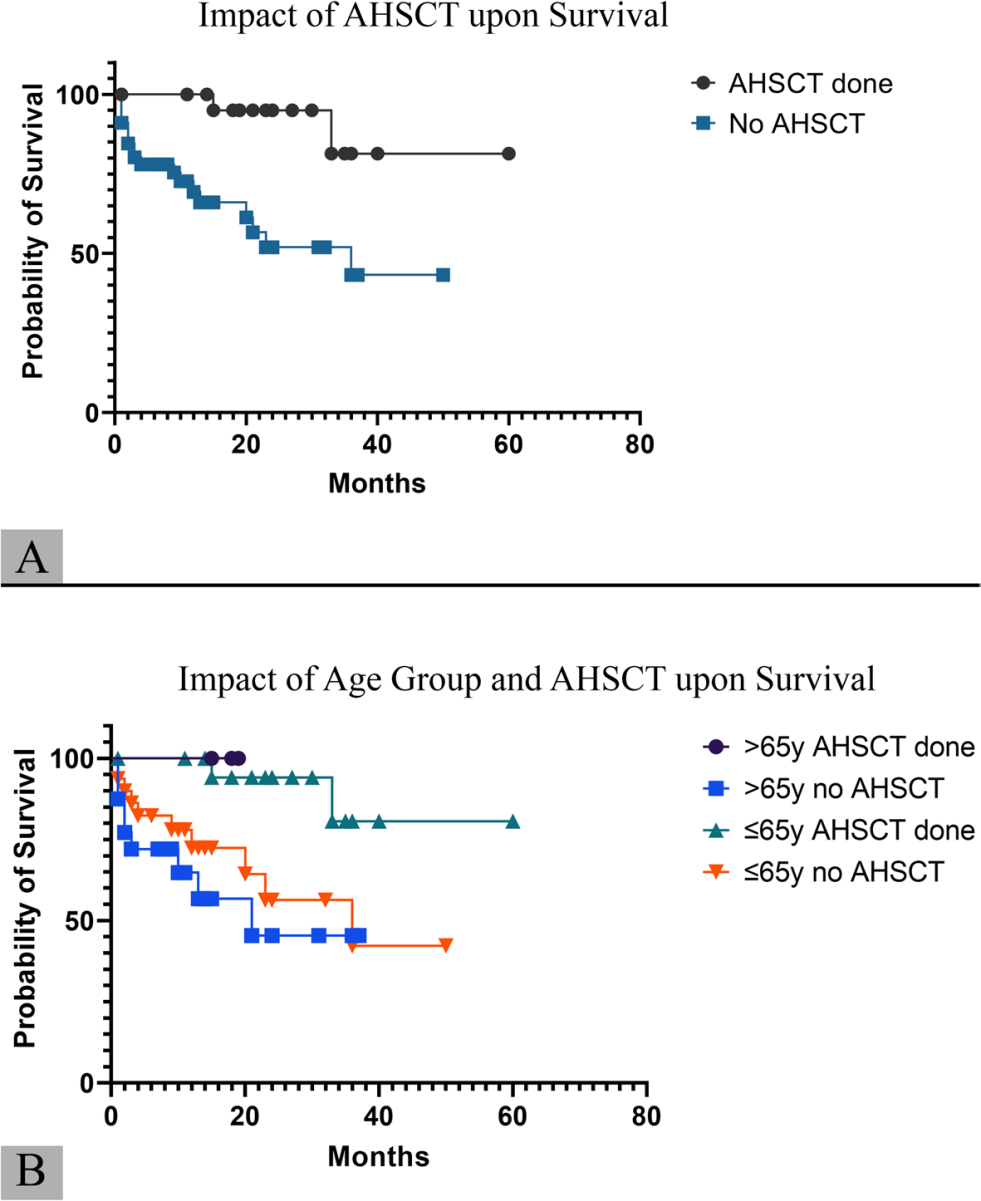
Significant difference (*p* 0.0023) in the survival curves of patients who underwent autologous hematopoietic stem cell transplant (AHSCT) and those who did not (**A**). Significant (*p* 0.0167) differences noted in the survival curves of patients aged above and up to 65 years of age, as per them having undergone AHSCT or not (**B**)

When the survival curves for those aged > 65 years versus those who were aged ≤ 65 years were compared, there was no significant difference in the survival (median OS not reached in both groups; p 0.0875) (Fig. 3). When the survival curves of four groups (aged > 65 years who underwent AHSCT, aged > 65 years who did not undergo AHSCT, aged ≤ 65 years who underwent AHSCT, and those aged ≤ 65 years who did not undergo AHSCT) were compared, there was a statistically significant difference favoring those who underwent AHSCT irrespective of the age group (*p* 0.0167) (Fig. 4).

## Discussion

The continuous addition of novel agents has led to the emergence of questions regarding the relevance of AHSCT in the treatment of MM. But in contrast to popular perception, the morbidity, mortality, and societal costs from non-transplant therapies are not negligible. The Mayo Stratification of Myeloma and Risk Adapted Therapy (mSMART) consensus statement has described the concept of TwiSTT (time without symptoms, treatment, and treatment toxicity). In addition to improving TwiSTT, AHSCT also spares the patient from the financial toxicity of newer novel agents which are currently more expensive than AHSCT [12–14].

Despite the valuable role of AHSCT in MM, it is rather a worldwide phenomenon that many transplant-eligible patients do not get the same. A study from Europe pooling 1802 patients revealed that 68.9% of eligible MM patients received an AHSCT. Another study specific to Eastern and Central Europe pooling 522 patients reported that 55.1% of eligible MM patients received an AHSCT [15, 16]. In the Indian scenario, it was reported by Nair et al. that only 26% of transplant-eligible myeloma patients underwent the procedure. The study observed that the most common reasons for eligible patients not undergoing transplants were fear (47%) and financial reasons (46%). The median age of patients who underwent transplants was lower than those who were not transplanted (52 years vs. 60 years) [17]. In comparison, 39.1% of the eligible MM patients in our series underwent an AHSCT, with the median age of transplanted patients in our series being lower compared to those who were not transplanted (53 years vs. 63 years). Another study from a major tertiary center in Southern India pooled 389 patients, of which only 23 (5.9%) underwent an AHSCT [18]. Though the percentage of patients who underwent transplants in our patient population is higher compared to prior data from India, the use of AHSCT is still limited when compared to the west. Interestingly though nearly 80% of patients aged more than 65 years were eligible for AHSCT only 14% of patients underwent the procedure. The common reasons implicated for poor transplant rates in the developing world include fear, poor affordability, and lack of access. However, one needs to consider physician bias about the tolerability of transplants in older patients. This bias was visible in our patient population. In the elderly patients though the majority were fit to undergo AHSCT only 29% of patients were offered the procedure. This bias may also stem from the limited inclusion of patients beyond 65 years old in clinical trials that studied AHSCT [19].

Frailty can be defined as a collective deterioration of multiple physical and physiological functions leading to a lower tolerance of stressors such as cancer and its treatments [20, 21]. Determination of fitness or frailty is an important challenge, especially to decide upon the intent and intensity of treatment among elderly patients with malignancy. However, there is currently no universally accepted method of frailty assessment. As an example, a systematic review by Buta et al. identified 70 different methods to define frailty [22].

Our study highlights the use of standardized fitness assessment tools in elderly myeloma patients. Of the numerous available fitness assessment tools, the CCI, the R-MCI, and the HSCT-CI are often utilized in trials involving MM patients. The International Myeloma Working Group (IMWG) frailty score was not used in our study since the same has not been validated for retrospective use [23]. The CCI has indeed been the most studied comorbidity scale and has been widely used in various contexts (including situations not involving malignant disorders). Studies using the CCI have assigned fitness as fit, intermediate fit, and unfit for those with 0, 1–2, or >2 points. The CCI classified 75.3% of the overall patient population in our dataset as being unfit for AHSCT. This index classified all patients >65years years as being unfit. Also, published literature reveals a low predictive value of the CCI for patients aged more than 40 years. Thus, despite having collected data, no further analysis was performed concerning CCI [10, 11, 24, 25]. The R-MCI is a myeloma-specific index that considers pulmonary function, renal function, the Karnofsky Performance Status, frailty, age, and unfavorable cytogenetics as input parameters. The maximum overall score is 9 points. Low, intermediate, and high risk are classified as per scores 0–3, 4–6, and 7–9, respectively. The maximum score for parameters such as renal function, lung function, frailty, and cytogenetics is 1 point each. However, the maximum score for Age and Karnofsky Performance status are 2 and 3 points, respectively. Thus, we hereby note that the R-MCI index is weighted in such a way that a patient with poor performance status and/or age >70 years could receive relatively higher R-MCI scores [26]. The HSCT-CI comprehensively comprises 17 different categories of organ dysfunction, but notably does not include age as an input parameter [27]. Nevertheless, in our series, both the HSCT-CI and the R-MCI demonstrated that at least 4 out of 5 patients aged beyond 65 years were AHSCT eligible. In our data set, the HSCT-CI classified a similar proportion of patients aged ≤ 65 years and > 65 years as transplant eligible, the R-MCI assigned a significantly lower number of patients aged ≤ 65 years as transplant-ineligible when compared to patients aged > 65 years. This could be attributed to the fact that the R-MCI considers age in years as an input parameter, assigning higher scores for those with advanced age.

The median age at diagnosis of MM is 54 years in India, which is a decade earlier compared to the West [28]. Most of the clinical trials utilizing AHSCT in myeloma have enrolled patients younger than 65 years of age. However, myeloma is mainly a disease of advanced age, and using a cut-off at 65 years would exclude a significant proportion of patients from the potential benefits of AHSCT. The same bias against older patients continues beyond clinical trials, and we identify it as another significant reason for the low rates of AHSCT among eligible MM patients. Patient eligibility for AHSCT is ideally done based upon overall health status which can be judged by using standardized fitness assessment tools as shown in our study. In contrast to results described by us and similar studies in India, data from advanced countries in the west show a different trend. Analysis of both the EBMT (European blood and marrow transplantation) and the CIBMR (center for international blood and marrow research) registries have shown a constant increase in the use of AHSCT among patients 65 and older from 1991 to 2010 [29, 30].

It has been reported that there was no difference in terms of treatment-related mortality (1%) for patients aged 60–65 years versus those aged beyond 65 years [31]. It has been argued that patients up to 80 years could be considered for AHSCT provided eligibility. Another study with a median age of 72 years concluded that satisfactory results could be expected with melphalan at a dose of 140mg/m^2^ [32]. In our study, patients who underwent transplants had better survival compared to patients who were not transplanted irrespective of age. More importantly, none of the transplanted patients aged more than age 65 years had significant morbidity or mortality due to transplant.

There has been a continued improvement in survival in MM with regard to early mortality and outcomes in older patients. Between 2001 and 2010, 1038 patients were grouped as two cohorts: those diagnosed between 2001 and 2005 and those diagnosed between 2006 and 2010. It was observed that the median OS for the 2001–2005 group was 4.6 years and for the 2006–2010 group was 6.1 years. The investigators importantly remarked that the improvement was primarily seen among patients over 65 years. The 6-year OS for those above 65 years of age was 31% and 56% for those in 2001–2005 versus the 2006–2010 group [33]. In a multicenter retrospective collaborative study of the Japanese society of myeloma and the European Myeloma network, it was noted that AHSCT helped enhance outcomes in the elderly. It has been remarked that “transplant eligibility” in itself is a prognostic factor [34] as seen in our study. Thus, it is safe to conclude that AHSCT is a safe and effective approach among fit, older patients with MM as seen in our study population, and transplant eligibility itself was also prognostic in our study.

Ours is a retrospective study involving a heterogeneous population, and it was not possible to elucidate the possible impact upon OS by factors such as treatment-related mortality (TRM), relapse, comorbidities, differences in induction and maintenance treatments, socioeconomic background, and others.

We acknowledge that “financial unaffordability” is an important factor leading to the low rates of AHSCT among eligible MM patients [35]. Low rates of AHSCT in eligible populations may also be due to false physician perception about patient fitness to undergo AHSCT as shown in our study.

## Conclusions

While non-affordability is indeed a factor causing low rates of transplants worldwide, other factors such as a bias against advanced age do indeed contribute. We conclude by stressing the importance of utilizing objective fitness assessment in every patient with MM. This could ensure that every fit patient enjoys the benefit of AHSCT, irrespective of age.

## Data Availability

The datasets used and/or analyzed during the current study are available from the corresponding author on reasonable request.

## Abbreviations

MM: Multiple myeloma
AHSCT: Autologous hematopoietic stem cell transplant
R-MCI: Revised-Myeloma Comorbidity Index
HSCT-CI: Hematopoietic Stem Cell Transplant-Comorbidity Index
LMIC: Low- and middle-income country
G-CSF: Granulocyte colony-stimulating factor
CCI: Charlson Comorbidity Index
OS: Overall survival
mSMART: Mayo Stratification of Myeloma and Risk Adapted Therapy
TwiSTT: Time without symptoms, treatment, and treatment toxicity
IMWG: International Myeloma Working Group
CIBMR: Center for international blood and marrow research
TRM: Treatment-related mortality

## References

[1] Auner HW, Garderet L, Kröger N. Autologous haematopoietic cell transplantation in elderly patients with multiple myeloma. Br J Haematol. 2015;171(4):453–62. 26213240 10.1111/bjh.13608

[2] Rosenberg AS, Brunson A, Jonas BA, Keegan THM, Wun T. Association between autologous stem cell transplant and survival among californians with multiple myeloma. J Natl Cancer Inst. 2019;111(1). [cited 2020 Oct 7]. Available from: https://pubmed.ncbi.nlm.nih.gov/29897481/. 10.1093/jnci/djy073 PMC6335109 29897481

[3] Jagannath S, Vesole DH, Zhang M, Desikan KR, Copeland N, Jagannath M, et al. Feasibility and cost-effectiveness of outpatient autotransplants in multiple myeloma. Bone Marrow Transplant. 1997;20(6):445–50. [cited 2020 Oct 7] Available from: https://pubmed.ncbi.nlm.nih.gov/9313876/10.1038/sj.bmt.1700900 9313876

[4] Clemmons AB, Anderegg S. Mixed outpatient-inpatient autologous stem cell transplant for multiple myeloma: a cost-saving initiative in a resource constrained environment. J Oncol Pharm Pract. 2017;23(5):384–8. [cited 2020 Oct 7]. Available from: https://pubmed.ncbi.nlm.nih.gov/27000280/. 10.1177/1078155216639753 27000280

[5] Rondelli D, Kun TL, Mathews V, Gooneratne LV, Tuladhar S, Neupane S, et al. First Global Blood & Marrow Transplant [GlobalBMT] Conference in Kathmandu with experiences from Nepal, India, Singapore and Sri Lanka. J Glob Health. 2018;8(1):1–5.

[6] Berro M, Arbelbide JA, Rivas MM, Basquiera AL, Ferini G, Vitriu A, et al. Hematopoietic cell transplantation–specific comorbidity index predicts morbidity and mortality in autologous stem cell transplantation. Biol Blood Marrow Transplant. 2017;23(10):1646–50. Available from: 10.1016/j.bbmt.2017.06.014. 10.1016/j.bbmt.2017.06.014 28669923

[7] Zweegman S, Engelhardt M, Larocca A. Elderly patients with multiple myeloma: towards a frailty approach? Curr Opin Oncol. 2017;29:315–21. 28763310 10.1097/CCO.0000000000000395

[8] Antoine-Pepeljugoski C, Braunstein MJ. Management of newly diagnosed elderly multiple myeloma patients. Curr Oncol Rep. 2019;21:1. [cited 2020 Oct 7]. Available from: https://pubmed.ncbi.nlm.nih.gov/31127403/. 10.1007/s11912-019-0804-4 31127403

[9] Marini C, Maia T, Bergantim R, Pires J, Aguiar E, Guimarães JE, et al. Real-life data on safety and efficacy of autologous stem cell transplantation in elderly patients with multiple myeloma. Ann Hematol. 2019;98(2):369–79. [cited 2020 Oct 7]. Available from: https://pubmed.ncbi.nlm.nih.gov/30368589/. 10.1007/s00277-018-3528-x PMC6342895 30368589

[10] Dold SM, Möller MD, Ihrost G, Langer C, Pönisch W, Mügge LO, et al. Validation of the revised myeloma comorbidity index and other comorbidity scores in a multicenter German study group multiple myeloma trial. Haematologica. 2021;106(3):875–80. 32414853 10.3324/haematol.2020.254235 PMC7928005

[11] Farina L, Bruno B, Patriarca F, Spina F, Sorasio R, Morelli M, et al. The hematopoietic cell transplantation comorbidity index (HCT-CI) predicts clinical outcomes in lymphoma and myeloma patients after reduced-intensity or non-myeloablative allogeneic stem cell transplantation. Leukemia. 2009;23(6):1131–8. 19194465 10.1038/leu.2009.1

[12] Martino M, Morabito F. Autologous stem cell transplantation in multiple myeloma is not dead but alive and well. Expert Opin Biol Ther. 2015;15(2):149–54. 25435056 10.1517/14712598.2015.988611

[13] Gonsalves WI, Buadi FK, Ailawadhi S, Bergsagel PL, Chanan Khan AA, Dingli D, et al. Utilization of hematopoietic stem cell transplantation for the treatment of multiple myeloma: a Mayo Stratification of Myeloma and Risk-Adapted Therapy (mSMART) consensus statement. Bone Marrow Transplant. 2019;54(3):353–67. Available from: 10.1038/s41409-018-0264-8. 29988062 10.1038/s41409-018-0264-8 PMC6463224

[14] Fermand J-P, Katsahian S, Divine M, Leblond V, Dreyfus F, Macro M, et al. High-dose therapy and autologous blood stem-cell transplantation compared with conventional treatment in myeloma patients aged 55 to 65 years: long-term results of a randomized control trial from the Group Myelome-Autogreffe. J Clin Oncol. 2005;23(36):9227–33. [cited 2020 Jul 16]. Available from: http://www.ncbi.nlm.nih.gov/pubmed/16275936. 10.1200/JCO.2005.03.0551 16275936

[15] Coriu D, Dytfeld D, Niepel D, Spicka I, Markuljak I, Mihaylov G, et al. Real-world multiple myeloma management practice patterns and outcomes in selected Central and Eastern European countries. Polish Arch Intern Med. 2018;128(9):500–11. [cited 2020 Jul 16]. Available from: https://pubmed.ncbi.nlm.nih.gov/30057386/. 10.20452/pamw.4305 30057386

[16] Raab MS, Cavo M, Delforge M, Driessen C, Fink L, Flinois A, et al. Multiple myeloma: practice patterns across Europe. Br J Haematol. 2016;175(1):66–76. [cited 2020 Jul 16]. Available from: https://pubmed.ncbi.nlm.nih.gov/27291397/. 10.1111/bjh.14193 27291397

[17] Nair CK, Selvaraj K, Raghavan V, A M, Shenoy PK, Kurup AR, et al. Limiting factors for autologous transplantation among transplant-eligible multiple myeloma patients: lesson from a Tertiary Cancer Centre in rural India. Leuk Res. 2019;83:106167. Available from: 10.1016/j.leukres.2019.106167 10.1016/j.leukres.2019.106167 31200146

[18] Jacob LA, Suresh Babu MC, Lakshmaiah KC, Govind Babu K, Lokanatha D, Rajeev LK, et al. Multiple myeloma: experience of an institute in limited resource setting. Indian J Cancer. 2017;54(1):340–2. [cited 2020 Jul 16]. Available from: https://pubmed.ncbi.nlm.nih.gov/29199718/. 10.4103/ijc.IJC_87_17 29199718

[19] Al Hamed R, Bazarbachi AH, Malard F, Harousseau JL, Mohty M. Current status of autologous stem cell transplantation for multiple myeloma. Blood Cancer J. 2019;9(4):44. 10.1038/s41408-019-0205-9. 10.1038/s41408-019-0205-9 PMC6453900 30962422

[20] Isaacs A, Fiala M, Tuchman S, Wildes TM. A comparison of three different approaches to defining frailty in older patients with multiple myeloma. J Geriatr Oncol. 2020;11:311–5. 31326393 10.1016/j.jgo.2019.07.004 PMC8161529

[21] Mccarthy AL, Peel NM, Gillespie KM, Berry R, Walpole E, Yates P, et al. Validation of a frailty index in older cancer patients with solid tumours. BMC Cancer. 2018;18:892. 30217171 10.1186/s12885-018-4807-6 PMC6137752

[22] Buta BJ, Walston JD, Godino JG, Park M, Kalyani RR, Xue QL, et al. Frailty assessment instruments: systematic characterization of the uses and contexts of highly-cited instruments. Ageing Res Rev. 2016;26:53–61. 26674984 10.1016/j.arr.2015.12.003 PMC4806795

[23] Palumbo A, Bringhen S, Mateos MV, Larocca A, Facon T, Kumar SK, et al. Geriatric assessment predicts survival and toxicities in elderly myeloma patients: an International Myeloma Working Group report. Blood. 2015;125(13):2068–74. 25628469 10.1182/blood-2014-12-615187 PMC4375104

[24] Shahjahan M, Alamo J, de Lima M, Khouri I, Gajewski J, Andersson B, et al. Effect of comorbidities on allogeneic hematopoietic stem cell transplant outcomes in AML/MDS patients in first complete remission. Biol Blood Marrow Transplant. 2004;10(Suppl 1):S12–3.

[25] Xhaard A, Porcher R, Chien JW, de Latour RP, Robin M, Ribaud P, et al. Impact of comorbidity indexes on non-relapse mortality. Leukemia. 2008;22(11):2062–9. 10.1038/leu.2008.197. 18685612 10.1038/leu.2008.197 PMC2637383

[26] Engelhardt M, Domm AS, Dold SM, Ihorst G, Reinhardt H, Zober A, et al. A concise revised Myeloma Comorbidity Index as a valid prognostic instrument in a large cohort of 801 multiple myeloma patients. Haematologica. 2017;102(5):910–21. 28154088 10.3324/haematol.2016.162693 PMC5477610

[27] Sorror ML, Maris MB, Storb R, Baron F, Sandmaier BM, Maloney DG, et al. Hematopoietic cell transplantation (HCT)-specific comorbidity index: a new tool for risk assessment before allogeneic HCT. Blood. 2005;106(8):2912–9. 15994282 10.1182/blood-2005-05-2004 PMC1895304

[28] Yanamandra U, Khattry N, Kumar S, Raje N, Jain A, Jagannath S, et al. Consensus in the management of multiple myeloma in india at myeloma state of the art 2016 conference. Indian J Hematol Blood Transfus. 2017;33(1):15–21. 28194051 10.1007/s12288-016-0773-9 PMC5280871

[29] Costa LJ, Zhang MJ, Zhong X, Dispenzieri A, Lonial S, Krishnan A, et al. Trends in utilization and outcomes of autologous transplantation as early therapy for multiple myeloma. Biol Blood Marrow Transplant. 2013;19(11):1615–24. [cited 2020 Jul 16]. Available from: https://pubmed.ncbi.nlm.nih.gov/23939198/. 10.1016/j.bbmt.2013.08.002 PMC3952066 23939198

[30] Auner HW, Szydlo R, Hoek J, Goldschmidt H, Stoppa AM, Morgan GJ, et al. Trends in autologous hematopoietic cell transplantation for multiple myeloma in Europe: increased use and improved outcomes in elderly patients in recent years. Bone Marrow Transplant. 2015;50(2):209–15. [cited 2020 Jul 16]. Available from: https://pubmed.ncbi.nlm.nih.gov/25387088/. 10.1038/bmt.2014.255 25387088

[31] Straka C, Liebisch P, Salwender H, Hennemann B, Metzner B, Knop S, et al. Autotransplant with and without induction chemotherapy in older multiple myeloma patients: long-term outcome of a randomized trial. Haematologica. 2016;101(11):1398–406. [cited 2020 Jul 16]. Available from: https://pubmed.ncbi.nlm.nih.gov/27662018/. 10.3324/haematol.2016.151860 PMC5394869 27662018

[32] Badros A, Barlogie B, Siegel E, Morris C, Desikan R, Zangari M, et al. Autologous stem cell transplantation in elderly multiple myeloma patients over the age of 70 years. Br J Haematol. 2001;114(3):600–7. 11552985 10.1046/j.1365-2141.2001.02976.x

[33] Kumar SK, Dispenzieri A, Lacy MQ, Gertz MA, Buadi FK, Pandey S, et al. Continued improvement in survival in multiple myeloma: changes in early mortality and outcomes in older patients. Leukemia. 2014;28(5):1122–8. 10.1038/leu.2013.313. 24157580 10.1038/leu.2013.313 PMC4000285

[34] Ozaki S, Harada T, Saitoh T, Shimazaki C, Itagaki M, Asaoku H, et al. Survival of multiple myeloma patients aged 65-70 years in the era of novel agents and autologous stem cell transplantation. A multicenter retrospective collaborative study of the Japanese Society of Myeloma and the European Myeloma Network. Acta Haematol. 2014;132(2):211–9. 10.1159/000357394. 24662986 10.1159/000357394

[35] Sharma SK, Choudhary D, Gupta N, Dhamija M, Khandelwal V, Kharya G, et al. Cost of hematopoietic stem cell transplantation in India. Mediterr J Hematol Infect Dis. 2014;6(1):e2014046. 25045454 10.4084/MJHID.2014.046 PMC4103507

